# Protective role of fatty acid oxidation against epithelial barrier dysfunction in allergic asthma

**DOI:** 10.1080/13510002.2026.2613534

**Published:** 2026-01-19

**Authors:** Muyun Wang, Yanan He, Haiyang Hu, Di Wu, Ximing Liao, Jing Gao, Shaoyong Gao, Huiming Yin, Kian Fan Chung, Qiang Li, Kun Wang, Wei Gao

**Affiliations:** a Department of Respiratory and Critical Care Medicine, Shanghai East Hospital, School of Medicine, Tongji University, Shanghai, People’s Republic of China; b National Heart and Lung Institute, Imperial College London, London, United Kingdom; c Department of Vascular Surgery, Shanghai Sixth People's Hospital Affiliated to Shanghai Jiao Tong University School of Medicine, Shanghai, People’s Republic of China; d Department of Respiratory and Critical Care Medicine, First Affiliated Hospital, Hunan University of Medicine, Huaihua, People’s Republic of China

**Keywords:** Bronchial asthma, Airway epithelial barrier, fatty acid oxidation, Carnitine palmitoyltransferase 1A, Oxidative stress, Etomoxir, L-carnitine, house dust mite

## Abstract

**Background:**

Fatty acid oxidation (FAO) is implicated in lung diseases, but its role in bronchial asthma is not fully understood. We investigated its effect on airway epithelial barrier integrity.

**Methods:**

Using a house dust mite (HDM)-induced murine asthma model and HDM, IL-4, IL-13, or TNF-α stimulated human primary bronchial epithelial cells (BECs) and bronchial epithelial (Beas-2b) cells, we modulated FAO with L-carnitine (agonist) and Etomoxir (inhibitor). BECs and Beas-2b cells were infected with lentivirus-mediated *CPT1A* shRNA prior to stimulation. Barrier function, mitochondrial oxidative stress, inflammation, and metabolism were assessed.

**Results:**

FAO level in lungs negatively correlated with increased inflammation and tissue injury in HDM-induced asthmatic mice (all *p* < 0.05), while positively regulating tight junction protein expression. In BECs and Beas-2b cells, Etomoxir treatment and CPT1A knockdown exacerbated the impairment of FAO caused by various stimulants (all *p* < 0.05). Furthermore, FAO negatively regulated HDM/cytokine-induced epithelial barrier damage, hyperactive inflammatory response, and mitochondrial dysfunction in Beas-2b cells (all *p* < 0.05). In contrast, treatment with L-carnitine significantly alleviated these pathophysiological features in both *in vivo* and *in vitro* models.

**Conclusion:**

FAO plays a protective role in the occurrence and development of asthma by maintaining airway epithelial cell homeostasis and barrier function.

## Background

Bronchial asthma (or asthma) is a heterogeneous inflammatory disease consisting of multiple phenotypes, affecting 5-20% of the global population [1], involving inflammatory cells such as eosinophils and airway structural cells such as the epithelium. Important pathophysiological features include chronic airway inflammation, reversible airflow obstruction, bronchial hyper-responsiveness, and airway wall remodelling [2]. Existing asthma medications, including bronchodilators and corticosteroids, remain the mainstay of asthma treatment [3], but around 5–10% of asthmatics remain poorly controlled on these treatments [4].

As the first line of defense against environmental insults such as allergens, pollutants, and infections, airway epithelial cells (AECs) play an important role in maintaining epithelial barrier integrity [5]. Inhaled allergens such as house dust mite (HDM) may activate downstream innate immune responses by secreting alarmins such as interleukin (IL)-25, IL-33, and thymic stromal lymphopoietin (TSLP) [6, 7]. IL-25 has been associated with increased airway and blood eosinophil numbers as well as elevated serum immunoglobulin E (IgE) levels [8], with activated eosinophil function and promotion of T helper type 2 (Th2) cell differentiation [9]. TSLP is associated with neutrophil and eosinophilic inflammation [10], and IL-33 activation of the inflammatory pathway promotes the accumulation of immunological cells in the lungs [11]. During this process, epithelial cells act as direct targets of proinflammatory cytokines [12, 13]. Th2 cytokines IL-4 and IL-13 cause AECs dysfunction in asthma [12, 13]. On the other hand, the Th1 cytokine, TNF-α, is also released during allergic inflammation and may lead to the production of proinflammatory mediators, airway hyperresponsiveness, and remodelling [14], as well as promoting concurrent neutrophil recruitment with the cytokine IL-17A [9], increasing asthma susceptibility and severity [15]. The resulting homeostatic disruption of AECs is accompanied by diminished cell–cell adhesion, increased intercellular space, and permeability, resulting in a compromised epithelial barrier [15].

Pulmonary cells use fatty acids, glucose, and amino acids as substrates to provide the energy required for their function [16]. However, when the metabolic balance is disrupted, the process of ‘metabolic reprogramming’ may lead to the development of associated disorders [17, 18]. Alterations in fatty acid (FA) metabolism may play a significant role in the pathophysiology of pulmonary illnesses, such as chronic obstructive pulmonary disease [19], acute lung injury (ALI) [20], and asthma [21]. The most common FA utilization method, FA oxidation (FAO), occurs largely in the mitochondria and is dependent on the activity of its enzymatic systems [22]. Thus, carnitine palmitoyltransferase (CPT) governs important steps in the FAO process [23, 24], and its primary isoform, CPT1A, is mainly found in the lung tissue [24, 25]. In a lipopolysaccharide – induced ALI murine model, impaired FAO-induced alveolar epithelial cell dysfunction contributed to the lung inflammatory response [20]. Al-Khami [26] et al. reported that allergic airway inflammation increases FAO in inflammatory cells to support the production of asthma-associated cytokines. Esteves [27] et al. reported that an increase in fatty acid consumption in asthmatic airway smooth muscle cells was linked to their proliferation. Because of these findings, we were interested in the potential role of FAO in metabolic perturbation effects on the epithelial barrier. Therefore, we investigated how FAO regulates epithelial cell function and its role in asthma.

## Methods

### Public transcriptomic data analysis

The normalized public transcriptomic datasets of induced sputum samples in asthma and healthy controls obtained from GSE137268 (https://www.ncbi.nlm.nih.gov/geo/query/acc.cgi?acc=GSE137268) were selected for analysis. The heatmap was performed using the pheatmap R package. The R packages involved in Gene Set Enrichment Analysis (GSEA) included clusterProfiler package (for enrichment analysis) and msigdbr package (gene set source).

### Establishment of HDM-Induced Asthma Murine Model

This study was conducted in compliance with the ARRIVE 2.0 guidelines.

Specific-pathogen-free (SPF) female C57BL/6 mice, aged 6–8 weeks, were purchased from Shanghai Laboratory Animal Co. Ltd., China. All mouse experiments were conducted according to the approved protocols of the Laboratory Animal Research Center Review Board, Tongji University, Shanghai (approval number: TJBB03721106) (Shanghai, China). The mice were acclimatized in an SPF-grade animal facility (temperature 22 ± 1°C, humidity 50 ± 5%, 12-hour light/dark cycle, with free access to food and water). The asthmatic murine model was induced by HDM as previously described [28].

Mice were anesthetized, and 50 μL saline dissolved in HDM (2 mg/mL) (GreerLaboratories, Lenoir, XPB91D3A2.5) or normal saline alone was applied to their nares with a micropipette 3 times per week for 4 weeks to induce lung inflammation.

A total of 18-20 h after the last HDM exposure, mice were euthanized via CO₂ asphyxiation (gradual displacement at 20% chamber volume/min) followed by cervical dislocation as a secondary confirmatory method, in accordance with AVMA Guidelines (2020).

Mice were randomly divided into four groups: (i) control unsensitized and challenged mice, *n* = 4; (ii) mice exposed to HDM extracts after PBS, *n* = 4; (iii) mice exposed to HDM extracts after Etomoxir (ETO, 1.5 mg/kg) (MedChemExpress, USA, HY-50202), *n* = 4; and (iv) mice exposed to HDM extracts after L-carnitine (LCA, 300 mg/kg) (MedChemExpress, USA, HY-B0399), *n* = 4. PBS, Etomoxi, or L-carnitine was administered intraperitoneally. Mice were excluded if they exhibited death from non-experimental causes during the mold-making period.

### Bronchoalveolar lavage fluid (Balf) collection for total and differential leukocyte counting

After anesthesia, Balf was collected through intratracheal injection of 800 μL ice-cold sterile PBS, followed by careful withdrawal. The Balf samples were centrifuged for 10 min at 140 × g at 4°C and the Balf supernatant was used for subsequent analysis. While the cells were re-suspended in PBS, the total number of cells was determined using a hemocytometer. The cells in the Balf were stained with hematoxylin and eosin (H&E). Differential leukocyte counts were determined by 2 blinded investigators who counted at least 200 cells per slide from coded samples (with group identifiers removed).

### Serum and Balf cytokine levels

Blood samples (0.5-0.8 mL) were collected by cardiac puncture. The cytokine levels in Balf supernatant and serum were measured using an ELISA kit. Cytokine detection covered several products, including IL-4 (Multisciences Biotech, Co., Ltd, China, EK204), TNF-α (Multisciences Biotech, Co., Ltd, China, EK282), IL-17A (Multisciences Biotech, Co., Ltd, China, EK217), and IgE (Abcam, UK, ab157718).

### Malondialdehyde (MDA) and Lactate dehydrogenase (LDH) assay

Mouse lung tissues were collected, and their MDA and LDH levels were measured using Malondialdehyde (MDA) Content Assay Kit (upgraded) (TBARS Method) (Beijing Solarbio Science & Technology Co., Ltd., China, BC6415) and Lacate Dehydrogenase (LDH) Activity Assay Kit (Beijing Solarbio Science & Technology Co., Ltd., China, BC0685), according to the manufacturer’s instructions. The absorbance was measured using an iMark microplate reader (Molecular Devices, Sunnyvale, CA, USA).

### Histopathological, immunologic analysis and transmission electron microscope (TEM) of mouse lung and bronchial tissue

In separate mice, the left lung, in which bronchoalveolar lavage was not performed, was dissected and fixed in 4% paraformaldehyde. The fixed lung tissue was then embedded in paraffin and sectioned into 5 μm slices, which were stained with Oil Red-O (Servicebio technology Co., Ltd, Wuhan, China, G1015) to assess lipid deposition, hematoxylin and eosin (H&E) to assess inflammation, and Periodic Acid-Schiff (PAS) to assess goblet cells. Additionally, the slides were immunostained with an anti-ZO-1(Abcam, UK, ab276131, 1:500) antibody. All images were captured using a Nikon Eclipse C1 microscope (Nikon, Japan), and the results were quantified using ImageJ software.

Samples (ultra-thin sections of mouse bronchial tissue) were prepared as described previously [28] and were photographed under a TEM (JEOL-1010, Jeol, Tokyo, Japan).

### Cell Culture and Lentivirus Transduction

Human primary bronchial epithelial cells (BECs) were purchased from iCell (iCell Bioscience Technology Co., Ltd., Shanghai, China), cultured in a Primary Epithelial Cell Culture System (iCell Bioscience Technology Co. Ltd., Primed-iCell-001), following the manufacturer’s protocol. Bronchial epithelial (Beas-2b) cells (Shanghai Institutes for Biological Sciences, China Academy of Science, Shanghai) were cultured in high-glucose Dulbecco's Modified Eagle's medium (Hyclone, Logan, UT, USA, SH30022.FS) supplemented with 10% fetal bovine serum (BioInd, Israel, HS0502F), 100 U/mL penicillin, and 100 μg/mL streptomycin (Invitrogen, USA, 15140122). A recombinant lentiviral vector of *CPT1A* shRNA with the target sequence GCTGAGCCATGAAGCTCTTAG was designed and constructed by Zorin (Shanghai, China). Cells were collected to determine the effect of shRNA using an immunoblotting assay.

In an *in vitro* model, BECs and Beas-2b cells were pretreated with ETO (100 nM) or LCA (100 μg/mL) for 2 h before being challenged with HDM (80 μg/mL), IL-4 (100 ng/mL) (PeproTech, USA, 96-200-04-50), IL-13 (100 ng/mL) (PeproTech, USA, 96-200-13-50), or TNF-α (100 ng/mL) (PeproTech, USA, 96-300-01A-50) for 6/12/24 h.

### Cell viability assay

Cell Counting Kit-8 (Dojindo, Kumamoto, Japan, CK04) was used to evaluate cell viability following the manufacturer's protocol. The absorbance was measured at 450 nm using an iMark microplate reader (Molecular Devices, Sunnyvale, CA, USA).

### Measurement of mitochondrial integrity and activity

Mitochondrial ROS-scavenging activity was evaluated using the Mitosox Green (Invitrogen, USA, M36006) assay following the manufacturer's protocol and was measured and recorded with the aid of a SpectraMax M5/M5e fluorescent plate reader (Molecular Devices, USA) at wavelengths of 488/510 nm.

### Quantitative real-time PCR (qRT-PCR)

Total Beas-2b cells RNA was extracted 6 h after stimulation and tested using the QuantStudio™ 6 Flex Real-Time System. qRT-PCR was performed using TB Green Premix Ex Taq (Rox) (Takara, Japan, RR420A). The 2^-△△Ct^ method was used to quantify relative mRNA expression. The specific primers used (BioTNT, China) were *lactate dehydrogenase A* (LDHA), *hypoxia-inducible factor-1 alpha* (HIF-1α), *glucose transporter* (GLUT), *low-density lipoprotein receptor* (LDLr), CD36, *Peroxisome proliferator-activated receptor alpha* (PPAR-α), *IL-8*, *IL-6*, *IL-18* and *β-actin* (Supplementary Table 1).

### Immunoblotting assay

CPT1A, dynamin-related protein 1 (DRP1), fission1 (FIS1), optic atrophy 1 (OPA1), mitofusin 2 (MFN2), NLRP3, Caspase-1, p-AMP-activated protein kinase (p-AMPK), CD36, GLUT, LDHA, ZO-1, Occludin, and β-actin were analyzed by immunoblotting. Primary antibodies used were anti-CPT1A (Abcam, UK, ab234111), anti-DRP1 (Cell Signaling Technology, CST, USA, 8570), anti-FIS1 (CST, 32525), anti-OPA1 (CST, 80471), anti-MFN2 (CST, 9482), anti-NLRP3 (CST, 15101), Anti-Caspase-1 (Abcam, ab179515), anti-p-AMPK (CST, 50081), anti-CD36 (CST, 14347), anti-GLUT (CST, 12939), anti-LDHA (CST, 2012), anti-ZO-1 (Abcam, ab276131), anti-occludin (Abcam, ab216327), and anti-β-actin (CST, 3700) at a dilution ratio of 1:1000. The secondary antibodies included Anti-rabbit IgG, HRP-linked Antibody (CST, 7074), and Anti-mouse IgG, HRP-linked Antibody (CST, 7076) at a dilution of 1:2000. The bands were visualized using a Tanon 5200 Multifunction Automated Chemiluminescence Imaging System (Tanon Biotechnology, China), and the images were analyzed using the ImageJ software.

### Measurement of epithelial permeability

Beas-2b cells were seeded in 24-well transwell inserts (Corning Costar, NY, USA, 3428) and allowed to grow until they were fully integrated into a single layer. Fluorescein isothiocyanate (FITC)-labelled dextran (4 kDa; Sigma-Aldrich, FD4-100MG) was added to the apical compartments (luminal side), following the manufacturer's protocol. Excitation and emission wavelengths were 490 and 520 nm, respectively.

### Determination of citrate, acetyl coenzyme A (CoA) and ATP

The levels of citrate, acetyl CoA, and ATP in BECs from various groups were evaluated using the Citrate Assay Kit (Abcam, UK, ab83396), PicoProbe Acetyl CoA Assay Kit (Abcam, UK, ab87546), and Luminescent ATP Detection Assay Kit (Abcam, UK, ab113849), following the manufacturer's protocol.

### Immunofluorescence staining of junctional protein

Beas-2b cells, with or without ETO/LCA and stimulation, were incubated with anti-ZO-1 antibodies (Abcam, ab221547) (dilution 1:500) at 4°C overnight, followed by incubation with FITC-labeled secondary antibodies (Beyotime, China, A0562) (dilution 1:500) for 30 min at 37°C. Nuclei were stained with 2-(4-Amidinophenyl)−6-indolecarbamidine dihydrochloride (DAPI, Beyotime, China, C1002). Images were visualized using a confocal microscope (TCS SP8, Leica, Germany).

### Statistical analysis

Data are reported as mean ± standard error mean (SEM), and *p* < 0.05 was considered significant. Statistical analyses were performed using the GraphPad Prism software (v9.0, GraphPad Software, Inc.). The normality of data distribution was assessed using the Shapiro–Wilk test. Experiments involving multiple comparisons were evaluated using parametric tests (one-way ANOVA followed by Bonferroni's post hoc test, or two-way ANOVA followed by Tukey's post hoc analysis) or non-parametric tests (Kruskal – Wallis test). Comparisons between two groups were performed using an unpaired Student's *t*-test.

## Results

### FAO negatively correlated with lung allergic inflammation and airway barrier damage in asthmatic mice

**Figure 1.**
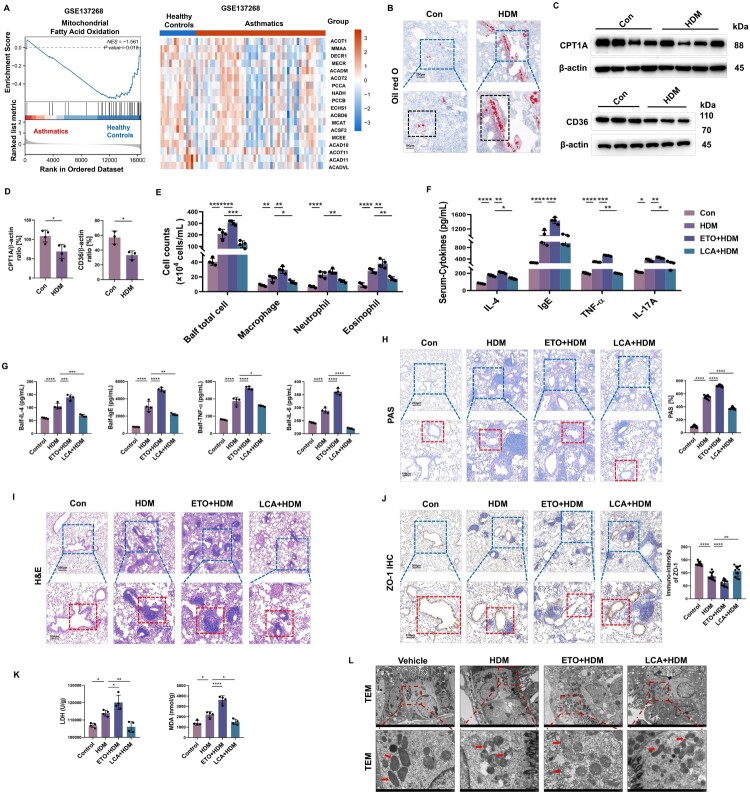
**FAO negatively correlated with lung injury in a chronic asthmatic mouse model. A** Public dataset GSE137268 was analyzed, and the genes related to mitochondrial fatty acid oxidation were selected and displayed in GSEA and heatmap analysis. **B** Representative pictures and a partial enlarged view of Oil red O-stained lung tissue sections. Scale bars in the upper panel, 200 μm; scale bars in the lower panel, 100 μm. The black dashed box shows the areas with significant changes. **C, D** Immunoblotting and its quantitative analysis identifying the CPT1A and CD36 expression in mice lungs. *n* = 3-4. **E** The counts of total inflammatory cells, macrophage, neutrophil, and eosinophils in Balf. *n* = 4. **F, G** Secretion of cytokines IL-4, IgE, TNF-α and IL-17A in Balf and serum were assessed by ELISA kits. *n* = 4. **H** Representative pictures and a partial enlarged view of PAS-stained lung tissue sections and was analyzed by ImageJ (*n* = 12). Scale bars in the upper panel, 200 μm; scale bars in the lower panel, 100 μm. The red dashed box shows the areas with significant changes. **I** Representative pictures and a partial enlarged view of H&E-stained lung tissue sections. Scale bars in the upper panel, 200 μm; scale bars in the lower panel, 100 μm. The red dashed box shows the areas with significant changes. **J** Immunohistochemical staining and partial enlarged view of ZO-1 protein were analyzed by ImageJ (*n* = 12). Scale bars in the upper panel, 200 μm; scale bars in the lower panel, 100 μm. The red dashed box shows the areas with significant changes. **K** Microplate reader assay exhibiting the production of MDA and LDH in lung tissue. *n* = 4. **L** Mitochondria in airway epithelial cells were determined by transmission electron microscopy (TEM) (Magnification×1500, 6000, respectively). Red arrow labeled mitochondrial. Data are presented as mean ± SEM, and *p* values were calculated with unpaired Student's *t*-test **D** or one-way ANOVA followed by Bonferroni's post-test or Kruskal–Wallis test. *p *<* *0.05*, **p *<* *0.01*, ***p *<* *0.001*, and ****p *<* *0.0001.*

We analyzed the GSE137268 dataset of transcriptomic data from induced sputum samples in asthma and healthy controls. By using gene set enrichment analysis (GSEA), we identified downregulated mitochondrial fatty acid oxidation metabolism and associated genes (Figure 1A). Based on this, we established a mouse asthma model using HDM and found that mice in the challenged group gathered more lipid droplets in their lungs, as shown by larger areas of Oil Red O dye accumulating in the lungs of the HDM group (Figure 1B), as well as reduced expression of mitochondrial fatty acid oxidation-related proteins CPT1A (reduced by 36.54%) and CD36 (reduced by 42.12%) (Figure 1C, D), indicating that HDM increased the amount of lipid droplets accumulated in mouse lungs, likely via suppression of FA consumption. FAO agonists and inhibitors were used to test this hypothesis. Balf inflammatory cell infiltration was increased in the HDM group, including total cell, macrophage, neutrophil, and eosinophil counts (all *p* < 0.05) (Figure 1E), with increased levels of IL-4, IgE, TNF-α, and IL-17A (all *p* < 0.05) in serum and Balf (Figure 1F, G). Compared with the PBS-treated HDM group, ETO further increased Balf cell counts by 22.44%, macrophages by 66.20%, neutrophils by 22.47%, and eosinophils by 35.71% (Figure 1E). IL-4, IgE, TNF-α, and IL-17A cytokine levels were further increased in Balf and serum (all *p* < 0.01) (Figure 1F, G). Conversely, upregulation of FAO by L-carnitine (LCA) effectively reversed the injury response induced by HDM. Asthmatic mice receiving LCA showed a reduction in airway inflammatory cells compared with PBS-treated mice, with a reduction across all cell types. Levels of IL-4, IgE, TNF-α, and IL-17A in Balf and serum were also reduced (all *p* < 0.05) (Figure 1E-G).

As shown in Figure 1H and I, HDM-exposed mice showed increased goblet cell metaplasia and mucus hypersecretion in the airways (all *p* < 0.0001) (Figure 1H). Lung histology revealed substantial airway inflammation in HDM-treated mice, with leukocytes congregating around the terminal bronchioles and capillary veins (Figure 1I). ETO-treated HDM mice showed increased inflammatory cell infiltration and greater goblet cell hyperplasia (*p* < 0.0001); however, these alterations were attenuated after LCA treatment (Figure 1H, I). PBS-treated HDM stimulation reduced the expression of the barrier-tight junction (TJ) protein ZO-1 in mouse lung tissues, particularly in AECs (Figure 1J). ETO-treated HDM mice showed a 30.22% reduction in ZO-1 expression in AECs compared with HDM alone, while LCA increased ZO-1 protein by 22.38% (Figure 1J). HDM stimulation increased the expression levels of MDA and LDH in mouse lung tissue, with a further elevation observed upon ETO intervention, indicating aggravated cellular damage and heightened oxidative stress, but asthmatic mice receiving LCA showed a reduction in MDA and LDH level (Figure 1K). In HDM-exposed mouse AECs, TEM analysis of mitochondria showed edema, vacuolation, along with broken or diminished cristae. ETO further exacerbated the damage to mitochondria, but following LCA pretreatment, mitochondrial integrity was restored, with reduced large-scale damage (Figure 1L).

### Modification of FAO regulates HDM-impaired AECs homeostasis and barrier function

**Figure 2.**
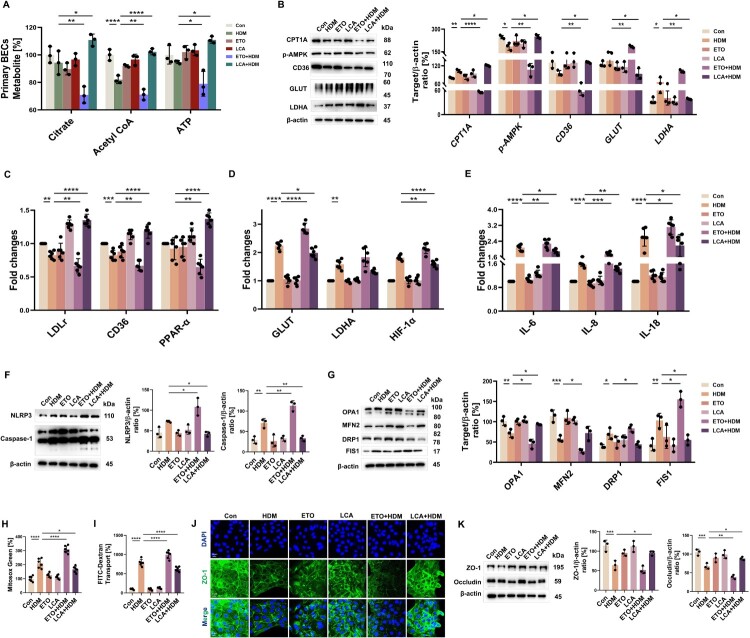
**FAO negatively regulates homeostasis and barrier function in AECs upon HDM challenge. A** After stimulated with ETO/LCA for 2 h and HDM for 12 h, levels of citrate, Acetyl CoA, and ATP in BECs were measured. *n* = 3. **B** Beas-2b cells stimulated with ETO/LCA for 2 h and HDM for 24 h, and immunoblotting and its quantitative analysis indicating the expression of CPT1A, p-AMPK, CD36, GLUT and LDHA. *n* = 3. Beas-2b cells stimulated with ETO/LCA for 2 h and HDM for 6 h, **C, D** Induction of LDLr, CD36, PPAR-α, GLUT, LDHA, and HIF-1α mRNA expression was estimated by qRT-PCR. Data are expressed as fold change. *n* = 6. **E** The induction of IL-6, IL-8, and IL-18 mRNA expression was estimated by qRT-PCR. Data are expressed as fold change. *n* = 6. Beas-2b cells stimulated with ETO/LCA for 2 h and HDM for 24 h, **F** Immunoblotting and its quantitative analysis indicating the expression of NLRP3 and Caspase-1. *n* = 3. **G** Immunoblotting and its quantitative analysis indicating the expression of OPA1, MFN2, DRP1 and FIS1. *n* = 3. **H** Microplate reader assay exhibiting the production of mitochondrial ROS. *n* = 6. **I** Microplate reader assay exhibiting FITC-Dextran Transport. *n* = 6. **J** Immunofluorescence indicating the expression of ZO-1. Scale bars, 20 μm. **K** Immunoblotting and its quantitative analysis indicating the expression of ZO-1 and Occludin. *n* = 3. Data are presented as mean ± SEM, and *p* values were calculated with one-way ANOVA followed by Bonferroni's post-test or Kruskal-Wallis test. *p *<* *0.05*, **p *<* *0.01*, ***p *<* *0.001*, and ****p *<* *0.0001.*

To assess the metabolic plasticity of AECs induced by exogenous stimulation, we examined the production of fatty acid metabolites in human primary BECs. The reduced expression of acetyl CoA, citrate, and ATP levels in BECs induced by HDM was intensified after ETO addition (reduced by 13.24%, 24.85%, and 16.73%, respectively), whereas it was rescued by LCA (increased by 24.78%, 17.57%, and 17.01%, respectively) (Figure 2A). Next, we verified whether mitochondrial function and metabolic states in HDM-challenged Beas-2b cells could be regulated by the inhibition and activation of FAO (the concentration of HDM was 80 μg/mL, the concentration of ETO was 100 nM, and the concentration of LCA was 100 μg/mL) (Supplementary Figure 1 A–C). As shown in Figure 2B–D, HDM exposure of these Beas-2b cells induced an unbalanced metabolic state with a decrease in the expression of FAO regulator and upregulated glycolysis regulator expression measured by immunoblotting and rt–PCR. Under the same HDM stimulation, CPT1A, p-AMPK, and CD36 protein expressions were further reduced by ETO (by 30.40%, 42.20%, and 41.05%, respectively), while glycolytic proteins GLUT and HIF-1α were increased by 34.76% and 52.90%, respectively. However, this HDM-deranged metabolism was reversed by LCA (all *p* < 0.05). Notably, LCA increased CPT1A protein levels in Beas-2b cells by 18.30% above basal levels. Metabolic regulator genes exhibited similar trends. The FAO regulator genes LDLr (0.83-fold), CD36 (0.81-fold), and PPAR-α (0.70-fold) were further reduced by 26.82%, 16.14%, and 20.39%, respectively, but the glycolysis regulator genes GLUT (1.27-fold), LDHA (1.16-fold), and HIF-1α (1.20-fold) were increased in ETO-treated HDM mice. However, the addition of LCA modulated metabolic gene expression to baseline levels (all *p* < 0.05) (Figure 2C, D).

Consistent with the *in vivo* experiment, the loss of FAO increased HDM-induced NLRP3/caspase-1 signaling pathway activation in Beas-2b cells (*p* = 0.0122 and *p* = 0.0013) (Figure 2F), including NLRP3 inflammasome activation, which is responsible for the secretion of IL-18 [29] as well as the proinflammatory cytokines IL-6 and IL-8 (all *p* < 0.05) (Figure 2E). In contrast, pretreatment with LCA suppressed inflammatory pathway activation (approximately 50%) as well as inflammatory cytokine IL-6, IL-8, and IL-18 levels (Figure 2E, F).

To determine whether FAO levels in Beas-2b cells prevented mitochondrial damage, mitochondrial reactive oxygen species (mtROS) levels following HDM stimulation were measured using Mitosox Green. The increase in mtROS induced by HDM could be exacerbated after ETO addition, indicating potential mitochondrial dysfunction and reduced mitochondrial ROS scavenging activity in ETO-treated Beas-2b cells (all *p* < 0.0001) (Figure 2H). Next, we examined the levels of mitochondrial fusion and fission protein expression and found that HDM did indeed cause mitochondrial structural impairment. Under HDM conditions, the levels of fusion proteins OPA1 and MFN2 decreased, whereas those of fission proteins DRP1 and FIS1 increased. OPA1 and MFN2 (reduced by 36.04% and 51.43%, respectively) and FIS1(increased by 50.65%) modifications were enhanced by ETO pretreatment (Figure 2G). LCA reduced the allergens generated mtROS (*p* = 0.0103), restored mitochondrial structural integrity by elevating OPA1 by 30.87%, and reduced DRP1 and FIS1 by 37.48% and 46.60%, respectively (Figure 2G, H).

We first used immunofluorescence labeling and confocal microscopy to analyze the intercellular junction structure. Control Beas-2b cells demonstrated predominant localization of ZO-1 in the areas of cell–cell contacts, which is indicative of an intact barrier (Figure 2J). The addition of ETO downregulated the decrease in ZO-1 expression induced by HDM; however, LCA rescued this barrier protein damage (Figure 2J). Immunoblotting analysis of total cell lysates also showed HDM stimulation caused a significant decrease in TJ proteins ZO-1 and Occludin (Figure 2K), with an increase in epithelial barrier function, as measured by the barrier leakage index FITC-dextran (Figure 2I). ETO caused a further decrease in protein levels (*p* = 0.0059) and increased permeability (*p* < 0.0001) caused by HDM. Pretreatment with LCA increased the barrier proteins ZO-1 and Occludin (*p* = 0.0195 and *p* = 0.047, respectively) and decreased cellular permeability (*p* = 0.0152). No measurable cellular cytotoxicity was induced by ETO or LCA treatment (Supplementary Figure 1D).

### CPT1A knockdown causes mitochondrial and barrier dysfunction of AECs upon HDM exposure

**Figure 3.**
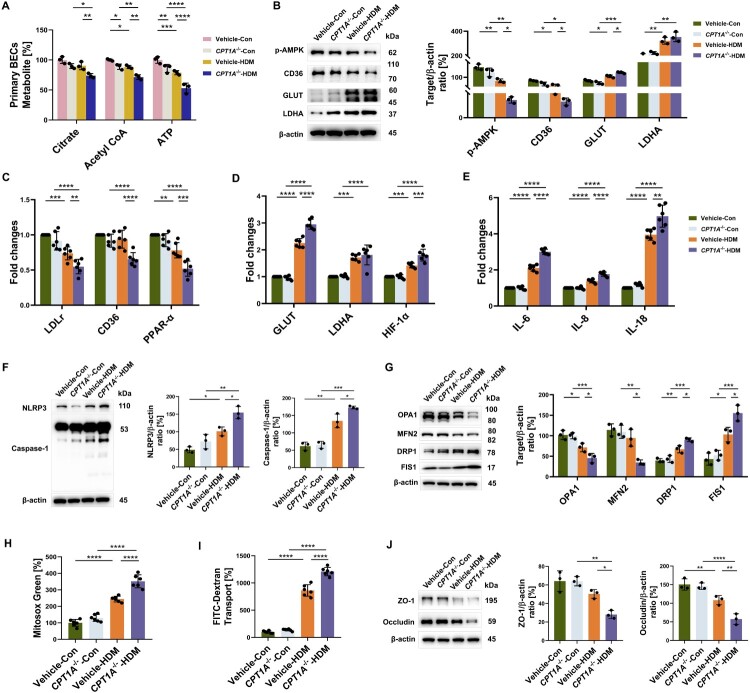
**CPT1A-FAO knockdown intensifies AECs and barrier damage upon HDM challenge. A** After stimulated with HDM for 12 h, levels of citrate, Acetyl CoA, and ATP in Vehicle/*CPT1A*^-/-^ – BECs were measured. *n* = 3. Vehicle/*CPT1A*^-/-^ – Beas-2b cells stimulated with HDM for 24 h, **B** Immunoblotting and its quantitative analysis indicating the expression of p-AMPK, CD36, GLUT and LDHA. *n* = 3. Vehicle/*CPT1A*^-/-^ – Beas-2b cells stimulated with HDM for 6 h, **C, D** Induction of LDLr, CD36, PPAR-α, GLUT, LDHA, and HIF-1α mRNA expression in cells was estimated by qRT-PCR. Data are expressed as fold change. *n* = 6. **E** The induction of IL-6, IL-8, and IL-18 mRNA expression in cells was estimated by qRT-PCR. Data are expressed as fold change. *n* = 6. Vehicle/*CPT1A*^-/-^ – Beas-2b cells stimulated with HDM for 24 h, **F** Immunoblotting and its quantitative analysis indicating the expression of NLRP3 and Caspase-1. *n* = 3. **G** Immunoblotting and its quantitative analysis indicating the expression of OPA1, MFN2, DRP1 and FIS1. *n* = 3. **H** Microplate reader assay exhibiting the production of mitochondrial ROS induced by HDM. *n* = 6. **I** Microplate reader assay exhibiting FITC-Dextran Transport induced by HDM. *n* = 6. **J** Immunoblotting and its quantitative analysis indicating the expression of ZO-1 and Occludin. *n* = 3. Data are presented as mean ± SEM, and *p* values were calculated with two-way ANOVA followed by Tukey post hoc analysis. *p *<* *0.05*, **p *<* *0.01*, ***p *<* *0.001*, and ****p *<* *0.0001.*

To explore the specific mechanisms underlying FAO damage in AECs, we knocked down CPT1A using *CPT1A* shRNA lentivirus in human primary BECs and Beas-2b cells. The reduction of CPT1A expression (Supplementary Figure 2A, B) caused a reduction in baseline levels of acetyl CoA by 11.28% and ATP by 14.07%, with citrate, acetyl CoA, and ATP further declining under HDM challenge (by 18.26%, 19.23%, and 32.60%, respectively) (Figure 3A). Simultaneously, compared with Vehicle cells, cell metabolic regulators in *CPT1A*^-/-^ –Beas-2b cells showed significant alterations after HDM exposure. The p-AMPK and CD36 protein levels were reduced, while GLUT protein was elevated (all *p* < 0.05), associated with the reduction of LDLr (0.74 fold), CD36 (0.70 fold), and PPAR-α (0.67 fold) genes, as well as further increased GLUT by 1.31-fold and HIF-1α by 1.27-fold (Figure 3B–D). There was a significant increase in the secretion of proinflammatory cytokines IL-18, IL-6, and IL-8 (all *p* < 0.01) (Figure 3E), as well as in HDM-induced NLRP3 (*p* = 0.0378) and Caspase-1 (*p* = 0.034) protein production in Beas-2b cells (Figure 3F).

*CPT1A*^-/-^ – cells exposed to HDM showed lower fusion protein and higher fission protein expression (all *p* < 0.05), with an increase in mtROS (*p* < 0.0001 compared with non-exposed cells) (Figure 3G, H). The barrier permeability index increased by 40.16% (Figure 3I), with a reduction in TJ protein ZO-1 and Occludin expression compared to the HDM-challenged Vehicle (*p* = 0.0298, and *p* = 0.0012) (Figure 3J).

### FAO relates negatively to AECs inflammatory response, oxidative stress and barrier dysfunction generated by Th1/Th2 cytokines

**Figure 4.**
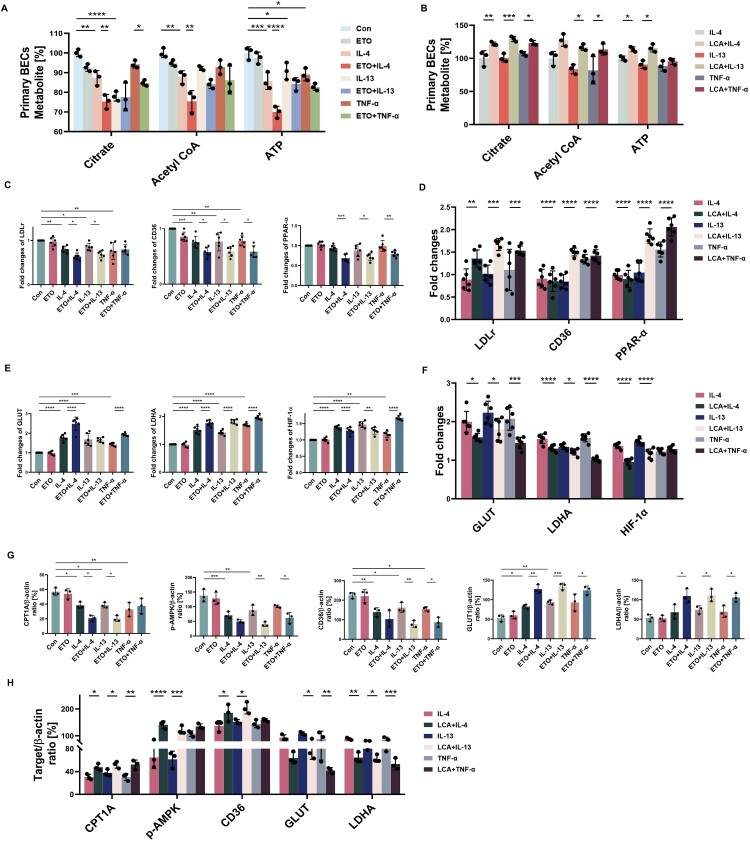
**Modification of FAO takes part in the metabolic status in Th1/Th2 challenged AECs. A, B** After being stimulated with ETO/LCA for 2 h and IL-4/IL-13/TNF-α for 12 h, levels of citrate, Acetyl CoA, and ATP in BECs were measured. *n* = 3. Beas-2b cells stimulated with ETO/LCA for 2 h and IL-4/IL-13/TNF-α for 6 h, **C-F** Induction of LDLr, CD36, PPAR-α, GLUT, LDHA, and HIF-1α mRNA expression was estimated by qRT-PCR. Data are expressed as fold change. *n* = 6. Beas-2b cells stimulated with ETO/LCA for 2 h and IL-4/IL-13/TNF-α for 24 h, **G-H** Quantitative immunoblotting analysis indicating the expression of CPT1A, p-AMPK, CD36, GLUT and LDHA. *n* = 3. Data are presented as mean ± SEM, and *p* values were calculated with one-way ANOVA followed by Bonferroni's post-test or Kruskal – Wallis test. *p *<* *0.05*, **p *<* *0.01*, ***p *<* *0.001*, and ****p *<* *0.0001.*

Both Th1 (TNF-α) and Th2 (IL-4/IL-13) cytokines (stimulation concentration of all cytokines was 100 ng/mL) caused a decrease in lipid metabolite content and ATP production in BECs, with a more pronounced effect in the presence of ETO, except for the effect of IL-13 (Figure 4A). In contrast, LCA alleviated the loss of citrate (increased by 21.97%, 28.62%, and 16.01%, respectively), acetyl CoA (33.09% higher than IL-13-exposed cells, and 31.49% higher than TNF-α-exposed cells), and ATP (13.78% higher than IL-4-exposed cells, and 24.87% higher than IL-13-exposed cells) in IL-4/IL-13/TNF-α stimulated BECs (Figure 4B). ETO addition to Beas-2b cells had a significant impact on LDLr, CD36, and PPAR-α (all *p* < 0.01) and GLUT, LDHA, and HIF-1α (all *p* < 0.05) mediator gene expression upon stimulation with optimal concentrations of these three cytokines (Supplementary Figure 3A-C) (Figure 4C, E). In addition, LCA enhanced LDLr, CD36, and PPAR-α (all *p* < 0.01) but reduced GLUT (0.80-, 0.81-, and 0.70-fold, respectively), LDHA (0.84-, 0.90-, and 0.65-fold, respectively), and HIF-1α (0.70-fold lower than IL-4-exposed cells, and 0.79-fold lower than IL-13-exposed cells), which were disrupted by cytokines (Figure 4D, F). As shown in Supplementary Fig. 4A and Figure 4G and H, the expression of CPT1A protein was significantly reduced under cytokine stimulation, which further declined with the addition of ETO, and was rescued by LCA (all *p* < 0.05). Th1 and Th2 cytokine-exposed cells contribute to a decrease in p-AMPK protein expression in FAO-limited cells. Moreover, in line with the qRT–PCR results, CD36 protein also further decreased with ETO addition, while GLUT and LDHA proteins increased (all *p* < 0.05).

**Figure 5.**
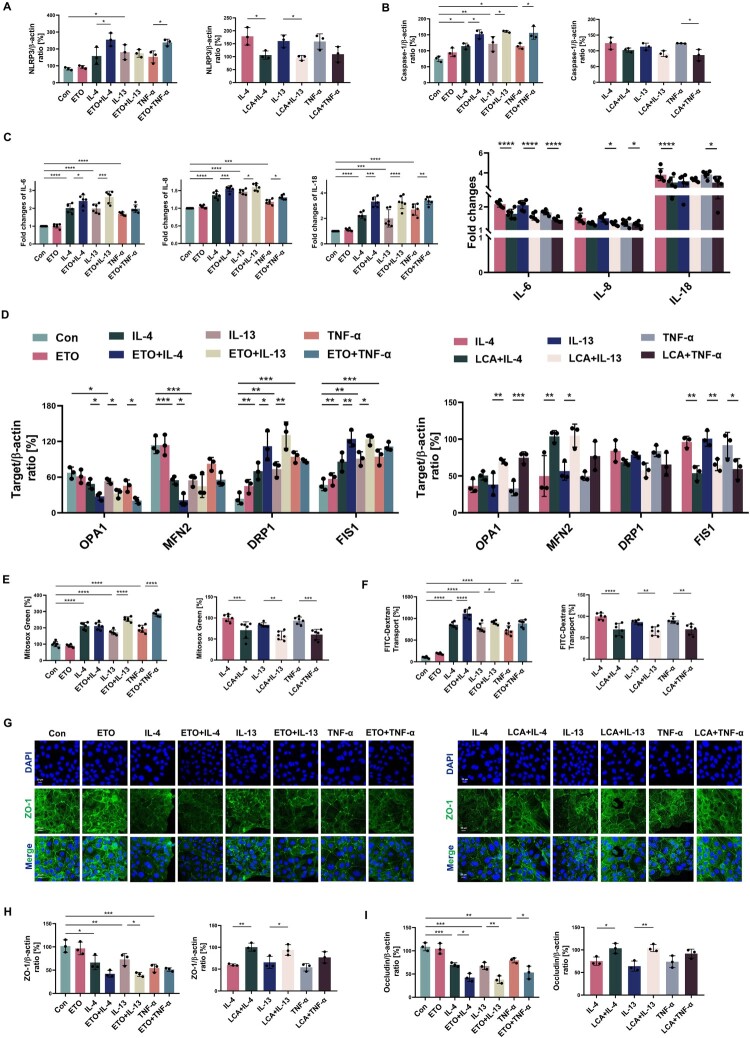
**FAO alteration is related to IL-4/IL-13/TNF-α induced inflammatory reaction, oxidative stress and epithelial barrier function. A, B** Beas-2b cells stimulated with ETO/LCA for 2 h and IL-4/IL-13/TNF-α for 24 h, quantitative immunoblotting analysis indicating the expression of NLRP3 and Caspase-1. *n* = 3. **C** Beas-2b cells stimulated with ETO/LCA for 2 h and IL-4/IL-13/TNF-α for 6 h, induction of IL-6, IL-8, and IL-18 mRNA expression in cells was estimated by qRT-PCR. Data are expressed as fold change. *n* = 6. Beas-2b cells stimulated with ETO/LCA for 2 h and IL-4/IL-13/TNF-α for 24 h, **D** Quantitative immunoblotting analysis indicating the expression of OPA1, MFN2, DRP1 and FIS1. *n* = 3. **E** Microplate reader assay exhibiting the production of mitochondrial ROS. *n* = 6. **F** Microplate reader assay exhibiting FITC-Dextran Transport. *n* = 6. **G** Immunofluorescence indicating the expression of ZO-1. Scale bars, 20 μm. **H, I** Quantitative immunoblotting analysis indicating the expression of ZO-1 and Occludin. *n* = 3. Data are presented as mean ± SEM, and *p* values were calculated with one-way ANOVA followed by Bonferroni's post-test or Kruskal – Wallis test. *p *<* *0.05*, **p *<* *0.01*, ***p *<* *0.001*, and ****p *<* *0.0001.*

As shown in Supplementary Figure 4B, Figure 5A-C, both Th1 and Th2 cytokines activated the NLRP3/caspase-1 pathway and promoted pro-inflammatory cytokine production (IL-6, IL-8, IL-18), which deteriorated when FA consumption was insufficient. ETO further intensified the activation of inflammasomes caused by IL-4, IL-13, and TNF-α, whereas LCA suppressed NLRP3 (*p* = 0.0126 compared with IL-4-exposed cells and *p* = 0.0251 compared with IL-13-exposed cells) and Caspase-1 (*p* = 0.0135) levels. Furthermore, ETO exacerbated mitochondrial dysfunction by increasing ROS release (all *p* < 0.0001) and structural degradation caused by Th1 or Th2 cytokines, including further imbalances in the expression of fusion (OPA1 and MFN2) and fission (DRP1 and FIS1) proteins (all *p* < 0.05) (Supplementary Figure 4C, Figure 5D, E). LCA significantly improved OPA1 (81.57% higher than IL-13-exposed cells, and 126.39% higher than TNF-α-exposed cells) and MFN2 (107.59% higher than IL-4-exposed cells, and 85.50% higher than IL-13-exposed cells) levels, and inhibited FIS1 (reduced by 44.07%, 35.31%, and 35.28%, respectively) in Beas-2b cells after stimulation, which maintained mitochondrial dynamics and further reduced ROS secretion (*p* = 0.0006, *p* = 0.0027, and *p* = 0.0001) (Figure 5D, E). The destructive effect of cytokine stimulation on the barrier protein ZO-1 was directly demonstrated by immunofluorescence images (Figure 5G), which could be positively regulated by FAO levels in Beas-2b cells. ETO exacerbated the damage of ZO-1 in Th1/Th2 cytokine-challenged cells, while LCA mitigated the loss of protein (Figure 5G). In addition, FAO showed a stronger effect on ZO-1 in cells challenged with IL-13 (*p* = 0.0154) in immunoblotting data (Supplementary Figure 4D, Figure 5H). Th1 and Th2 cytokines, in the absence and presence of ETO, decreased occludin protein (all *p* < 0.05) and increased barrier permeability (all *p* < 0.01) (Supplementary Figure 4D, Figure 5F-I). Furthermore, LCA restabilized the barrier against external stimuli, with an increase in the barrier protein ZO-1 (*p* = 0.0021 compared with IL-4-exposed cells, and *p* = 0.0254 compared with IL-13-exposed cells) and occludin (*p* = 0.0257 and *p* = 0.0022) to diminish barrier permeability (*p* < 0.0001, *p* = 0.0031, and *p* = 0.0025) (Figure 5F-I). These changes cannot be explained by any cellular cytotoxicity induced by ETO or LCA treatment (Supplementary Figure 5A-C).

### CPT1A and its regulated FAO knockdown exacerbates cytokines induced mitochondrial impairment and barrier function disorder in AECs

**Figure 6.**
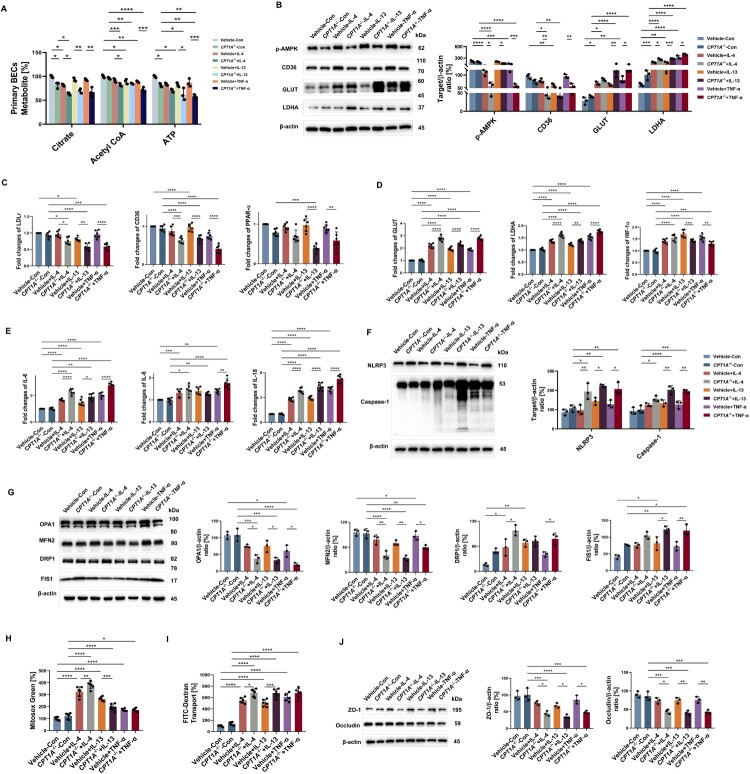
**FAO altered by CPT1A knockdown induces barrier damage upon IL-4/IL-13/TNF-α challenge, through influencing inflammation and mitochondrial damage.** **A** After stimulated with IL-4/IL-13/TNF-α for 12 h, levels of citrate, Acetyl CoA, and ATP in Vehicle/*CPT1A*^-/-^ BECs were measured. *n* = 3. **B** Vehicle/*CPT1A*^-/-^ Beas-2b cells stimulated with IL-4/IL-13/TNF-α for 24 h, and immunoblotting and its quantitative analysis indicate the expression of p-AMPK, CD36, GLUT and LDHA. *n* = 3. Vehicle/*CPT1A*^-/-^ Beas-2b cells stimulated with IL-4/IL-13/TNF-α for 6 h, **C, D** Induction of LDLr, CD36, PPAR-α, GLUT, LDHA, and HIF-1α mRNA expression was estimated using qRT-PCR. Data are expressed as fold change. *n* = 6. **E** The induction of IL-6, IL-8, and IL-18 mRNA expression was estimated using qRT-PCR. Data are expressed as fold change. *n* = 6. Vehicle/*CPT1A*^-/-^ – Beas-2b cells stimulated with IL-4/IL-13/TNF-α for 24 h, **F** Immunoblotting and its quantitative analysis indicate the expression of NLRP3 and Caspase-1. *n* = 3. **G** Immunoblotting and its quantitative analysis indicating the expression of OPA1, MFN2, DRP1 and FIS1. *n* = 3. **H** Microplate reader assay exhibiting the production of mitochondrial ROS induced by IL-4/IL-13/TNF-α. *n* = 6. **I** Microplate reader assay exhibiting FITC-Dextran Transport induced by IL-4/IL-13/TNF-α. *n* = 6. **J** Immunoblotting and its quantitative analysis indicating the expression of ZO-1 and Occludin. *n* = 3. Data are presented as mean ± SEM, and *p* values were calculated with two-way ANOVA followed by Tukey post hoc analysis. *p *<* *0.05*, **p *<* *0.01*, ***p *<* *0.001*, and ****p *<* *0.0001.*

Compared to Vehicle cells, *CPT1A*^-/-^ –primary BECs produced less citrate, acetyl-CoA, and ATP when exposed to the cytokines TNF-α, IL-4, and IL-13 (all *p* < 0.05) (Figure 6A). *CPT1A*^-/-^ –Beas-2b cells, when activated with IL-4, IL-13, and TNF-α (Figure 6B), showed reduced p-AMPK (by 46.87%, 22.20%, and 57.25%, respectively) and CD36 (by 43.93%, 29.91%, and 40.50%, respectively) levels, accompanied by increased GLUT and LDHA levels. LDLr (0.79, 0.72, and 0.61 fold, respectively), CD36 (0.73, 0.70, and 0.54 fold, respectively) and PPAR-α (0.75, 0.40, and 0.65 fold, respectively) gene expression were also reduced with greater expression of GLUT, LDHA and HIF-1α (all *p* < 0.01) in *CPT1A*^-/-^ –Beas-2b cells (Figure 6C, D). The RNA expressions of IL-6 and IL-18 (Figure 6E), and protein levels of NLRP3 (increased by 99.20%, 55.92%, and 61.56%, respectively) and Caspase-1 (increased by 25.74%, 52.06%, and 55.47%, respectively) were increased in cytokines treated *CPT1A*^-/-^ –Beas-2b cells (Figure 6F), compared with Vehicle ones.

Compared with Beas-2b cells treated with Vehicle, cells with lentivirus-mediated *CPT1A* shRNA showed lower levels of mitochondrial fusion proteins OPA1 and MFN2 (all *p* < 0.05). IL-4 and TNF-α increased DRP1 protein levels by 69.90% and 89.91%, respectively, whereas IL-13 and TNF-α increased FIS1 protein levels by 49.94% and 64.69%, respectively (Figure 6G). However, only the Th2 cytokines IL-4 and IL-13 increased mitochondrial ROS accumulation in *CPT1A*^-/-^ – Beas-2b cells (all *p* < 0.01) (Figure 6H).

Moreover, Th1 and Th2 cytokines reduced the expression of TJ proteins ZO-1 (*p* = 0.05, *p* = 0.0371, and *p* = 0.0158, respectively) and occludin (*p* = 0.14, *p* = 0.0046, and *p* = 0.006, respectively) expression (Figure 6J). Although all cytokines generated higher barrier permeability, only IL-4 and IL-13 enhanced the leakage in *CPT1A*^-/-^ – Beas-2b cells (all *p* < 0.05) (Figure 6I).

## Discussion

We demonstrated that the capacity of fatty acid consumption was reduced in the lungs of HDM-challenged mice, and that FAO levels were negatively correlated with the mouse lung inflammatory response and positively correlated with tight junction protein ZO-1 expression in airway epithelial cells. In addition, *in vitro* experiments on AECs showed that FAO was negatively correlated with the inflammatory effect and disrupted mitochondrial homeostasis in HDM and Th1/Th2 cytokine-challenged Beas-2b cells, which also interfered with epithelial cell permeability. These findings indicate that stimulation of airway epithelial cells with HDM and cytokines such as TNF-α, IL-4, and IL-13 led to metabolic changes, particularly decreased FAO utilization, which rapidly triggered a cellular inflammatory response, ultimately disrupting the stability of the epithelial barrier.

We showed that FAO plays a vital role in the pathogenesis of allergy-induced asthmatic mice. The mechanism by which HDM or Th1 or Th2 cytokines may cause airway epithelial barrier dysfunction has been the focus of our investigation. First, we tested the fatty acid metabolic content (acetyl CoA and citrate) and ATP generation in human primary BECs and determined whether the exogenous stimulus effect on AECs mainly affects FA utilization. To determine whether fatty acid metabolic changes have a direct effect on the epithelial barrier, we investigated the biological role of FAO in Beas-2b cells on cellular physiological events. By modulating FAO levels in cells by ETO and LCA, we unveiled the pivotal role of FAO in maintaining cell homeostasis and barrier integrity against HDM challenge. Knockdown of CPT1A in Beas-2b cells had an effect similar to that of the FAO inhibitor on bronchial epithelial dysfunction. CPT1A and its regulated FAO (CPT1A-FAO) knockdown enhanced HDM-induced Beas-2b cells and epithelial barrier injury, suggesting the importance of CPT1A in sustaining cellular FAO. We hypothesized that epithelial effects on asthma occur not only in the early stages but also throughout its progression, implying that the resulting inflammatory response (Th1/Th2 cytokines) generated by the injured epithelium exacerbates epithelial barrier dysfunction. Under Th1 (TNF-α) or Th2 (IL-4, IL-13) cytokine challenge, there were significant fluctuations in the FA metabolic levels in Beas-2b cells. Similarly, FAO is related to reduced inflammatory processes and mitochondrial dysfunction in cells. Notably, we found that cytokine-induced cell damage might also be regulated by CPT1A.

Asthma pathophysiology is regulated by immune cells that communicate closely with airway structural cells, such as AECs and bronchial smooth muscle (BSM) cells [24, 27]. In patients with asthma, there is evidence of metabolic reprogramming, which may be central to epithelial-mediated inflammatory responses [29]. Moreover, docosahexaenoic acid [30] and 1,25-dihydroxy vitamin D3 [31] have been reported to restore cellular healing and dysfunctional airway epithelial barriers in dust-activated bronchial epithelial cells by interfering with lipid metabolism. In our study, we found that the capacity for fatty acid consumption was reduced in the lungs of HDM-challenged mice, and the cytokine data implied an extensive downstream Th2 immune response, a process motivated by alarmins released by AECs, in the presence of the FAO inhibitor ETO. Therefore, lipid metabolism could alleviate allergen-induced AEC-immune cell crosstalk in asthma and improve the mucosal barrier of AECs. The mucosal barrier is governed by the integrity of intercellular junctions that connect AECs to one another (i.e. tight junctions, adherens junctions, and desmosomes), which eventually shut off the paracellular space [32]. Increased barrier leakage impairment enhances the access of pathogens and other insults to submucosal cells [6], resulting in more severe lung injury and exacerbating the asthmatic process and inflammation. External stimulation of AECs led to their metabolic reprogramming, particularly altering CPT1A-FAO utilization, which rapidly triggered a cellular inflammatory response, ultimately affecting the stability of the epithelial barrier. Targeting disruption of the epithelial barrier evoked by metabolic abnormalities may have broad implications in the management of asthma.

We further explored the specific mechanisms by which FAO lacks impaired mitochondrial integrity and metabolic patterns *in vitro*. Under normoxic conditions, the PPAR family is an important transcription factor for FAO [30, 31], and together with CPT1A, LDLr, and CD36, could influence FA metabolism by regulating intracellular free FA levels. HIF-1α [32, 33] and HIF-target genes (GLUT and LDH) [32, 34] may also promote glycolysis. By assessing these molecules, we found that HDM or cytokines caused increased glycolysis and decreased FAO. Consistent with our previous research that cells deprived of the FAO pathway would switch to more glycometabolism to maintain homeostasis [35], we discovered that AECs under exogenous stimulation would reduce FAO-related metabolites and ATP production levels while enhancing cell retention of glycolysis regulators, representing the cellular metabolic reprogramming caused by the imbalance of mitochondrial metabolic patterns. In addition to energy and biosynthetic intermediates, metabolic pathways regulate the equilibrium between ROS generation and antioxidant activity [36, 37]. In our study, through augmented mtROS release and the disequilibrium of fission and fusion [38, 39], external stimuli exacerbated the oxidative stress response and mitochondrial dynamic changes in CPT1A-FAO-deficient cells. Excessive intracellular ROS function as a second messenger [40, 41] and have been linked to asthma development by increasing mucus hypersecretion, airway hyperresponsiveness, and epithelium damage [42]. This also explains why FAO-deficient cells cause a higher inflammatory response and damage to the epithelial barrier under stimulation. Esteves et al. [27] discovered a causal relationship between FA metabolism and asthmatic BSM cell growth by identifying increased mitochondrial respiration. The disparity between our investigations might be mainly attributed to the differences in cell types and their roles in illness. Bronchial remodelling is a critical component of asthma pathogenesis and is a poor prognostic indicator in this disease. The most crucial result of BSM remodelling is increased bulk, a process that has been associated with increased mitochondrial biogenesis and mass [43]. This provides us with the possibility that the regulation of mitochondrial metabolic patterns (glucose and lipid metabolism) can be used as a preventive strategy, while taking advantage of the differences between various cell types to achieve targeted regulation and intervention for precise treatment.

Reinke et al. [21] supported our conclusion from a clinical perspective by identifying decreased fatty acid metabolism and β-oxidation in the sputum of patients with severe asthma. Their findings motivated us to expand existing research to investigate potential new targets for asthma therapy. Under cigarette smoke challenge, LCA has been reported to protect FAO in lung epithelial cells, thereby avoiding lung damage and eventual emphysema [44, 45]. Therefore, we introduced LCA as an FAO agonist to the simulation model. Pretreatment with LCA considerably improved the lung phenotype of HDM-induced animals while also improving TJ protein stability. LCA in AECs effectively improved CPT1A protein expression and subsequent FA metabolism, which maintained mitochondrial function balance and mitochondrial dynamin stability in cells, and critically restored the integrity of the epithelial barrier. Our work fills a gap by demonstrating that increasing AECs FAO levels might be a potential way to restore the asthmatic inflammatory process.

In summary, we confirmed impaired FAO function in an HDM-induced asthmatic murine model. Using FAO inhibition or activation, we concluded that FAO protects against lung goblet cell hyperplasia, mucus hypersecretion, and inflammation. However, FAO deficiency can lead to epithelial barrier dysfunction. Using primary BECs and Beas-2b cells, CPT1A-FAO was demonstrated to maintain superior barrier stability, secondary to its influence on mitochondrial regulatory and anti-inflammatory effects on HDM or Th1/Th2 cytokine-activated cells, which is a critical link in the progression of asthma. The FAO agonist L-carnitine causes the recovery of mitochondrial dysfunction and metabolism. Thus, downregulation of FAO in AECs increases the risk of developing asthma through disruption of epithelial metabolism and barrier function. Our study opens up the potential for novel treatments of asthma by activating FAO.

## Supplementary Material

Supplementary_Information_clean.docx

## Data Availability

The original contributions presented in the study are included in the article/supplementary material, and further inquiries can be directed to the corresponding authors.
