# Supplementary material for: Protective role of fatty acid oxidation against epithelial barrier dysfunction in allergic asthma
Source: Redox Rep. 2026 Jan 19;31(1):2613534. doi: 10.1080/13510002.2026.2613534 (PMC12821354; doi:10.1080/13510002.2026.2613534)
Supplement: Supplementary_Information_clean.docx [file YRER_A_2613534_SM4714.docx]

**Supplementary Information for**

**Protective role of fatty acid oxidation against epithelial barrier dysfunction in allergic asthma**

Muyun Wang^1,#^, Yanan He^1,#^, Haiyang Hu^3,#^, Di Wu^1^, Ximing Liao^1^, Jing Gao^1^, Shaoyong Gao^1^, Huiming Yin^4^, Kian Fan Chung^2^, Qiang Li^1^, Kun Wang^1,*^, Wei Gao^1,*^

1 Department of Respiratory and Critical Care Medicine, Shanghai East Hospital, School of Medicine, Tongji University, Shanghai 200092, China

2 National Heart and Lung Institute, Imperial College London, London SW3 6LY, United Kingdom

3 Department of Vascular Surgery, Shanghai Sixth People's Hospital Affiliated to Shanghai Jiao Tong University School of Medicine, Shanghai 200233, China

4 Department of Respiratory and Critical Care Medicine, First Affiliated Hospital, Hunan University of Medicine, Huaihua 418000, China

^#^ These authors contributed equally: Muyun Wang, Yanan He, Haiyang Hu.

*Corresponding author

Dr. Wei Gao* Department of Respiratory and Critical Care Medicine, Shanghai East Hospital, School of Medicine, Tongji University, No.150 Jimo Road, Pudong, Shanghai, P.R. China.

E-mail: grace19881118@126.com

Dr. Kun Wang* Department of Respiratory and Critical Care Medicine, Shanghai East Hospital, School of Medicine, Tongji University, No.150 Jimo Road, Pudong, Shanghai, P.R. China.

E-mail: Dr_Wangk@tongji.edu.cn

**Running title**: FAO modulates epithelial barrier dysfunction in asthma

**Supplementary Table 1 The sequences of qRT-PCR primers used in this study.**

|  | **Sequence (5' to 3')** | **Species** | **Accession No.** |
| --- | --- | --- | --- |
| *LDHA* forward | ATGGAGATTCCAGTGTGCCTGT | Human | NM_001165415 |
| *LDHA* reverse | CAGAGAGACACCAGCAACATTC | Human | NM_001165415 |
| *HIF-1α* forward | GAAACTTCTGGATGCTGGTGATTT | Human | NM_001243084 |
| *HIF-1α* reverse | GCAATTCATCTGTGCTTTCATGTCA | Human | NM_001243084 |
| *SLC2A1 (GLUT1)* forward | CTTCCAGTATGTGGAGCAACTGT | Human | NM_006516 |
| *SLC2A1 (GLUT1)* reverse | GCACAGTGAAGATGATGAAGACG | Human | NM_006516 |
| *CD36* forward | GGCTGTGACCGGAACTGTG | Human | NM_001001548 |
| *CD36* reverse | AGGTCTCCAACTGGCATTAGAA | Human | NM_001001548 |
| *PPAR-α* forward | CGTGCTTCCTGCTTCATAGATAAG | Human | NM_001393946 |
| *PPAR-α* reverse | GTGGTAGCGCTGGTCTAC | Human | NM_001393946 |
| *LDLr* forward | GTGTCACAGCGGCGAATG | Human | NM_001195800 |
| *LDLr* reverse | CGCACTCTTTGATGGGTTCA | Human | NM_001195800 |
| *CXCL8 (IL-8)* forward | TTTTGCCAAGGAGTGCTAAAGA | Human | NM_000584 |
| *CXCL8 (IL-8)* reverse | AACCCTCTGCACCCAGTTTTC | Human | NM_000584 |
| *IL-6* forward | ACTCACCTCTTCAGAACGAATTG | Human | NM_000600 |
| *IL-6* reverse | CCATCTTTGGAAGGTTCAGGTTG | Human | NM_000600 |
| *IL-18* forward | TCTTCATTGACCAAGGAAATCGG | Human | NM_001562 |
| *IL-18* reverse | TCCGGGGTGCATTATCTCTAC | Human | NM_001562 |
| *ACTB (β-actin)* forward | ACCGAGCGCGGCTACA | Human | NM_001101 |
| *ACTB (β-actin)* reverse | CAGCCGTGGCCATCTCTT | Human | NM_001101 |


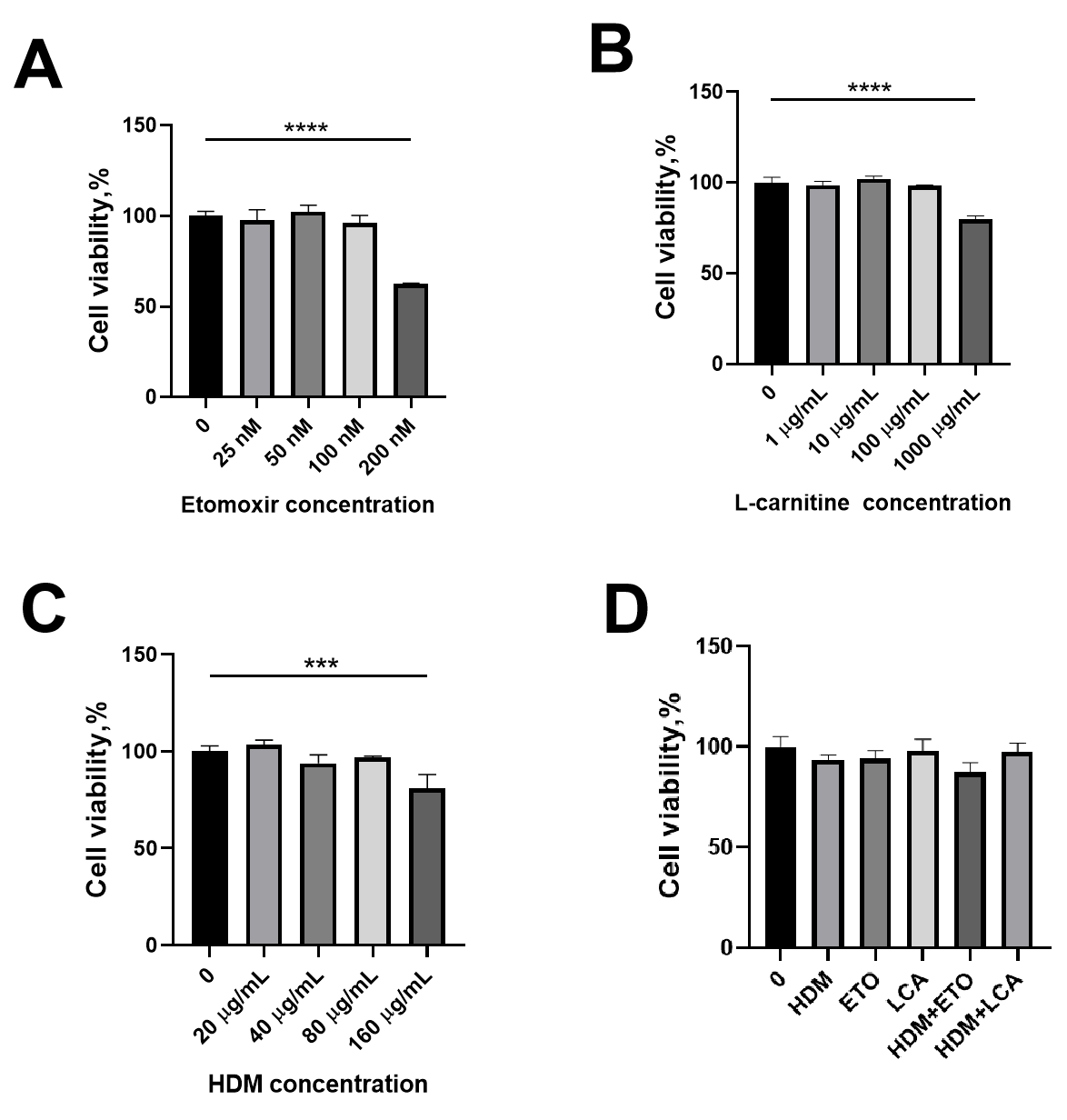


**Supplementary Fig. 1** Cell viability of Beas-2b cells with different doses of **A** Etomoxir, **B** L-carnitine, **C** HDM, respectively, for 24 hours. **D** Cell viability of Beas-2b cells treated with HDM for 24 hours, with/without 2-hour-ETO/LCA. Data are presented as mean ± SEM and *p* values were calculated with one-way ANOVA followed by Bonferroni's post-test. ****p < 0.001*, and *****p < 0.0001.*


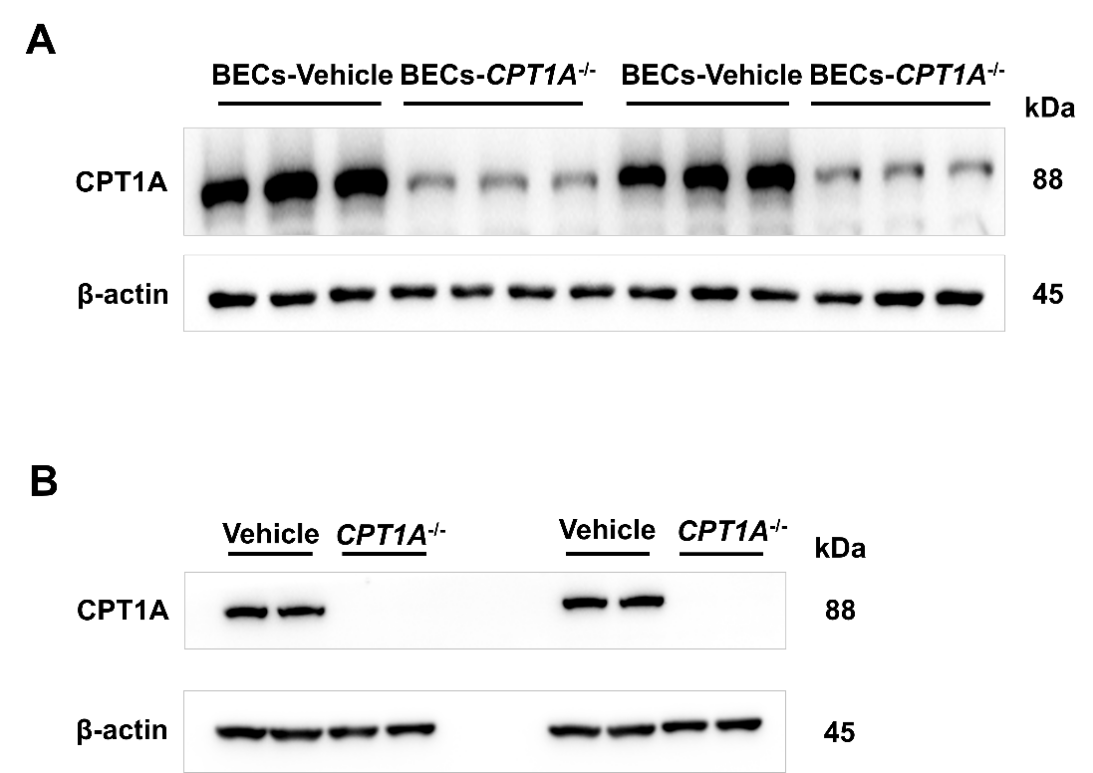


**Supplementary Fig. 2 A** Immunoblotting indicating CPT1A expression in BECs infected with lentivirus-mediated shRNA (*CPT1A*^-/-^) or negative control shRNA (Vehicle). *n* = 6. **B** Immunoblotting indicating CPT1A expression in Beas-2b cells infected with lentivirus-mediated shRNA (*CPT1A*^-/-^) or negative control shRNA (Vehicle). *n* = 4.

**
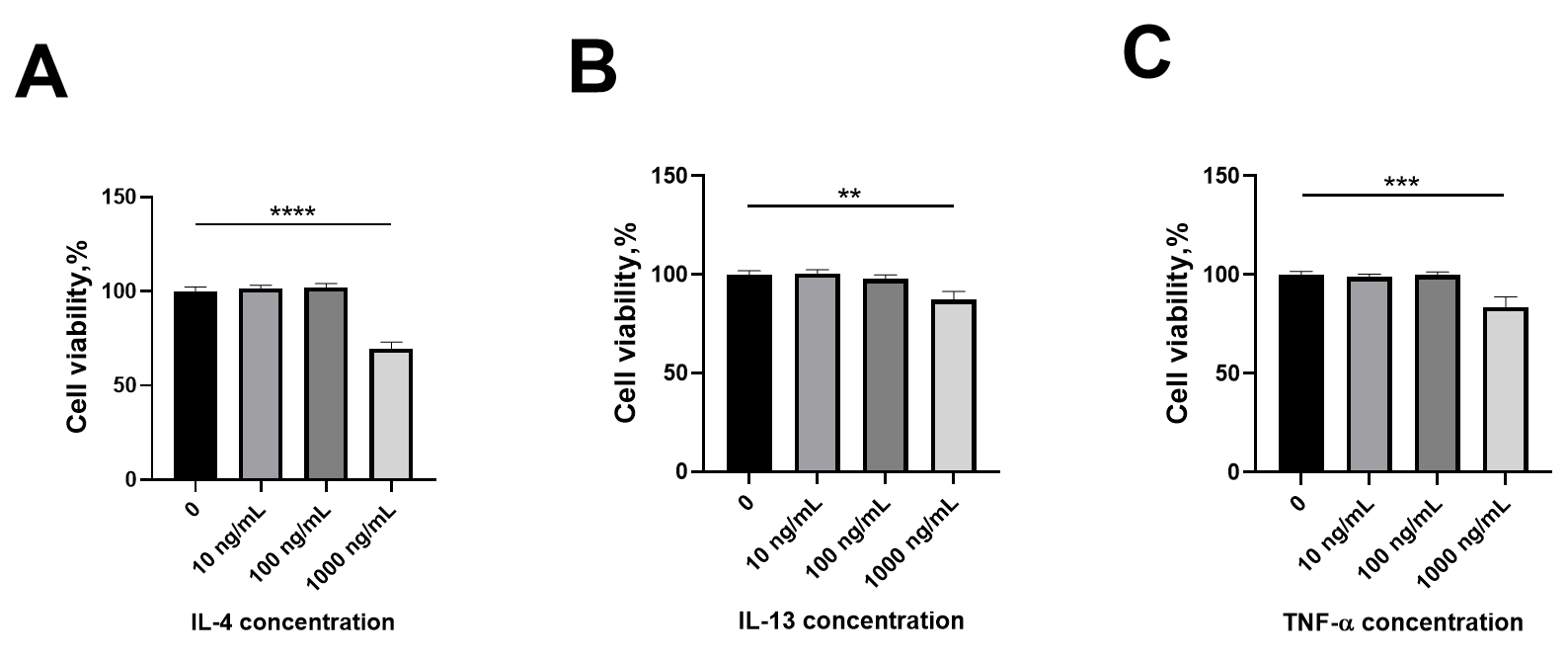
**

**Supplementary Fig. 3** Cell viability of Beas-2b cells with different doses of **A** IL-4, **B** IL-13, and **C** TNF-α, for 24 hours, respectively. Data are presented as mean ± SEM and *p* values were calculated with one-way ANOVA followed by Bonferroni's post-test. ***p < 0.01*, ****p < 0.001*, and *****p < 0.0001.*


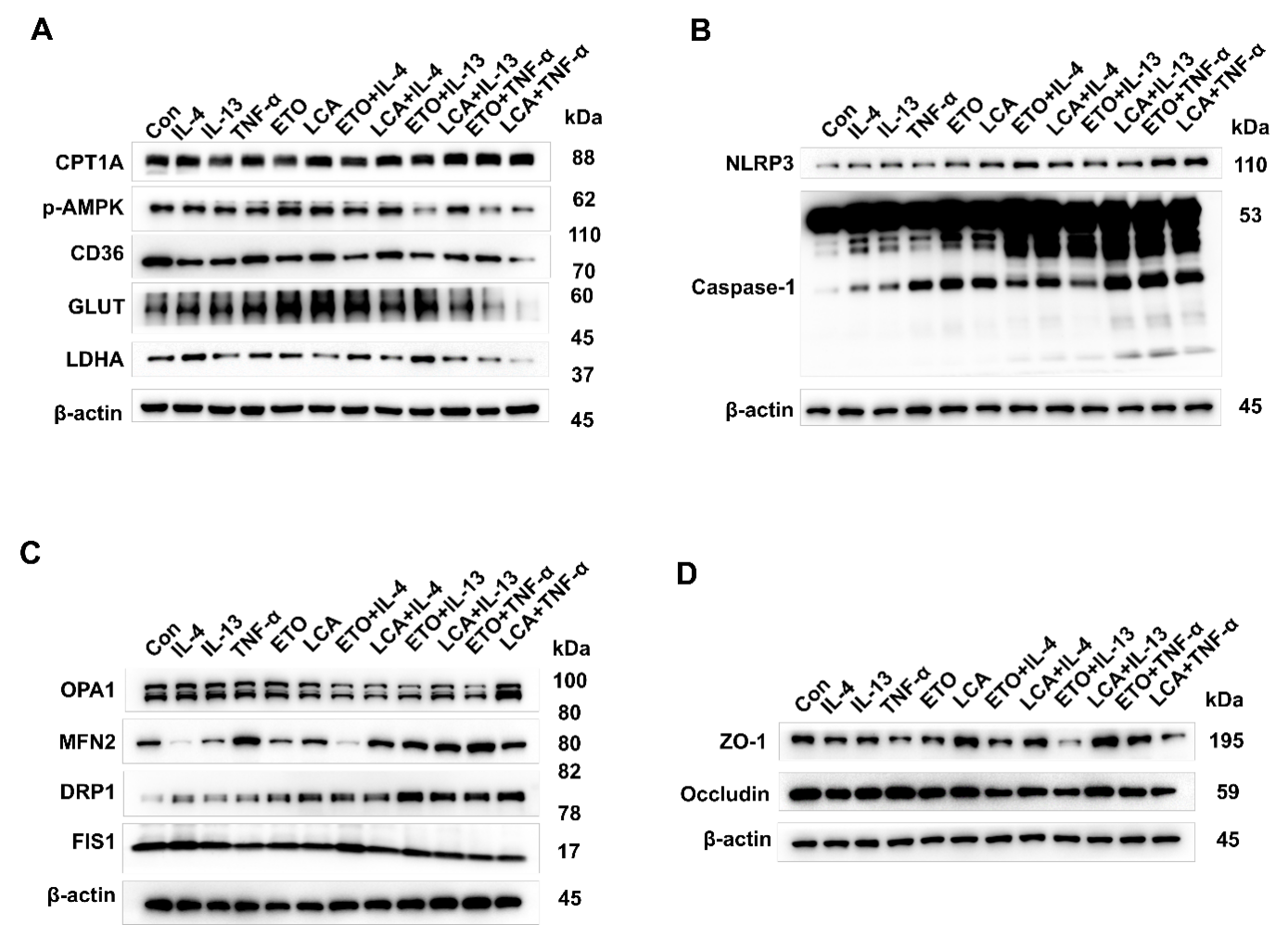


**Supplementary Fig. 4 A** Immunoblotting assay demonstrating the regulatory effects of IL-4/IL-13/TNF-α with or without ETO/LCA on CPT1A, p-AMPK, CD36, GLUT, LDHA and β-actin expression in Beas-2b cells. **B** Immunoblotting assay demonstrating the regulatory effects of cytokines with or without ETO/LCA on NLRP3, Caspase-1 and β-actin expression in Beas-2b cells. **C** Immunoblotting assay demonstrated the regulatory effects of IL-4/IL-13/TNF-α with or without ETO/LCA on OPA1, MFN2, DRP1, FIS1 and β-actin expression in cells. **D** Immunoblotting assay demonstrating the regulatory effects of cytokines with or without ETO/LCA on ZO-1, Occludin and β-actin expression in Beas-2b cells.


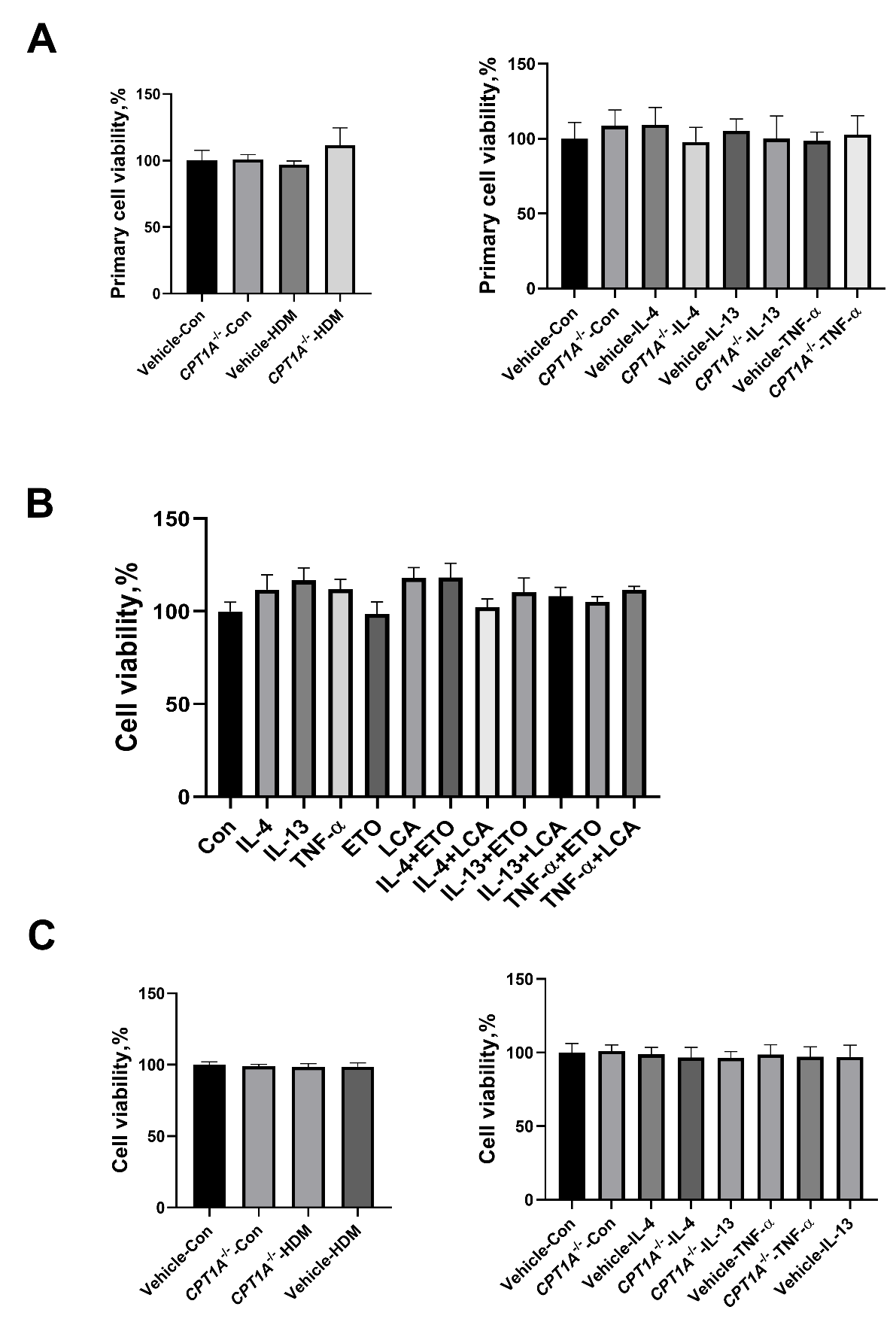


**Supplementary Fig. 5 A** Cell viability of Vehicle and *CPT1A*^-/-^ BECs treated with HDM, and IL-4/IL-13/TNF-α, for 24 hours, respectively. **B** Cell viability of Beas-2b cells treated with IL-4/IL-13/TNF-α for 24 hours, with/without 2-hour-ETO/LCA. **C** Cell viability of Vehicle and *CPT1A*^-/-^ Beas-2b cells treated with HDM, and IL-4/IL-13/TNF-α for 24 hours, respectively. Data are presented as mean ± SEM and *p* values were calculated with one-way ANOVA followed by Bonferroni's post-test **B** or two-way ANOVA followed by Tukey post hoc analysis.
